# Clinical efficacy of flap transfer coverage in the treatment of vesicovaginal fistula

**DOI:** 10.1007/s00192-023-05465-w

**Published:** 2023-02-04

**Authors:** Xingqi Wang, Zhenhua Gao, Ling Li, Daoming Tian, Hang Zhou, Jihong Shen

**Affiliations:** 1 Department of Urology, The First Affiliated Hospital of Kunming Medical University, Kunming, 650032 China; 2 Yunnan Province Clinical Research Center for Chronic Kidney Disease, Kunming, 650032 China

**Keywords:** Vesicovaginal fistula, Transvaginal approach, Flap transfer coverage, Surgical technique, Retrospective analysis

## Abstract

**Introduction and hypothesis:**

Vesicovaginal fistula (VVF) brings severe psychological, physiological, and social stress to patients, which seriously affects the quality of their sexual life. Traditional transvaginal repair surgery can cause vaginal shortening. Transferring the lateral free flap can maintain vaginal length. This study was carried out to investigate the clinical efficacy of the surgery of flap transfer coverage for treating VVF.

**Methods:**

A retrospective analysis was performed on 37 patients diagnosed with VVF and repaired by flap transfer coverage in the Urogynecology department of the First Affiliated Hospital of Kunming Medical University from January 2018 to June 2021. All patients took a prone split leg position to repair VVF with the flap transfer covering method and a chart review was performed.

**Results:**

Among the 37 patients, there were 34 cases of primary complete healing, and the success rate reached 91.89% without recurrence and complications. Three cases recurred with leakage of urine; cystoscopy showed that the fistula was significantly reduced, and all patients were cured after secondary repair by the same surgical method without complications.

**Conclusions:**

Flap transfer coverage is a safe and effective surgical method for repairing VVF. The prone split leg position can better increase exposure. The fistula being away from the incision suture is the key to the success of the operation. Transferring the fistula can effectively improve the cure rate of VVF. Transferring the lateral free flap can maintain vaginal length.

**Supplementary information:**

The online version contains supplementary material available at 10.1007/s00192-023-05465-w

## Introduction

Vesicovaginal fistula (VVF) is the most common fistula in the genitourinary tract. It is due to an abnormal anatomical connection between the bladder and the vagina that causes involuntary continuous or unremitting urinary incontinence [1]. Long-term urinary leakage brings severe psychological, physiological, and social stress to patients [2], which seriously affects the quality of the sexual life of patients. In developed countries, the main cause of VVF is iatrogenic injury caused by pelvic surgery such as hysterectomy or radiotherapy for pelvic tumors. Hillary et al. [3], in their systematic review, reported that 75% of VVFs occurred after abdominal hysterectomy. However, VVFs in developing countries are mostly due to prolonged labor and long-term obstructive delivery, resulting in ischemic necrosis and the subsequent development of a VVF [4, 5]. With the continuous improvement of medical and health levels, especially the current large number of laparoscopic developments, more and more delayed fistulas and apical fistulas have emerged because of excessive electrocautery. It has become a hot topic for pelvic floor doctors, and also is heavy burden for gynecologists. A retrospective analysis was performed on 37 patients diagnosed with VVF and repaired by transvaginal flap transfer coverage in the Urogynecology department of the First Affiliated Hospital of Kunming Medical University from January 2018 to June 2021. This study explored the clinical efficacy of the surgery of transvaginal flap transfer coverage for treating VVF.

## Patients and methods

### Patients

All 37 patients in this group were admitted to the hospital with continuous or intermittent urine leakage from the vagina. The patients and fistula characteristics are shown in Table 1. Patients’ age ranged from 30 to 61 years (average 49 years). The body mass index was 21.1 to 25.8 kg/m^2^ (average 24.3 kg/m^2^. The course of disease ranged from 2 weeks to 24 months (average 6.5 months). There were 6 cases of obstetric fistula among them, including 1 case of fistula after vaginal delivery and 5 cases of fistula after caesarean section. Twenty-eight cases of gynecological fistula were all post-hysterectomy, with no history of pelvic radiotherapy. There were 3 cases of urological fistula, including 2 cases after radical resection of bladder cancer and 1 case after urinary incontinence. Four patients had a history of surgical repair of VVF. This study was approved by the Ethics Committee of the First Affiliated Hospital of Kunming Medical University (IRB No.[2022] Ethics Review L No.33, 27 April 2022), and the patients were contacted and agreed to follow-up examinations.

**Table 1:** Characteristics of patients and fistulas

	Total(*N* = 37)
Age (years)	49 (30–61)
BMI (kg/m^2^)	24.3 (21.1–25.8)
Duration (months)	6.5 (0.5–24)
Obstetric fistula	6 (16.2%)
Vaginal delivery	1 (2.7%)
Caesarean section	5 (13.5%)
Gynaecological fistula (post-hysterectomy)	28 (75.7%)
Urological fistula	3 (8.1%)
Radical resection of bladder cancer	2 (5.4%)
Surgery for urinary incontinence	1 (2.7%)
Primary fistula	33 (89.2%)
Secondary fistula	4 (10.9%)
Fistula number	
Single	32 (86.5%)
Multiple	5 (13.5%)
Fistula size	0.4–5.0 cm

### Preoperative evaluation

Urethral catheterization with a 16-Fr catheter is performed to drain the bladder and the methylene blue dye test was performed to further confirm the diagnosis. All patients underwent urinalysis and urine culture examination, indicating different degrees of urinary tract infection, and select sensitive antibiotic anti-infective therapy before surgery. Cystoscopic examination was used to confirm the presence of the VVF, determined the presence of additional fistulas, and assessed size, number, and location in relation to the ureteric orifices. When the opening of the fistula is unidentifiable, passage of a guidewire through the fistula may help identification. Preoperative intravenous urography was carried out in all patients to rule out ureterovaginal fistulas [1]. In this group, there were 37 patients with 32 cases of a single fistula and 5 cases of multiple fistulas. The fistula is 0.4 to 5 cm in diameter and more than 0.5 cm from the ureteral orifice. If the fistula is close to the ureter, the ureteral stent can be inserted to avoid damage to the ureter during surgery. The perineum and vaginal cavity are scrubbed with iodophor or normal saline 3 days before surgery, and then estrogen ointment is routinely applied daily to the vaginal cavity.

It is necessary to assess the patient's sexual life before surgery. The choice of the free flap transfer method depends on the patient's sexual activity. Transferring the lateral free flap for sexually active patients can maintain vaginal length and avoid vaginal shortening.

### Surgical procedure

**Fig. 1:**
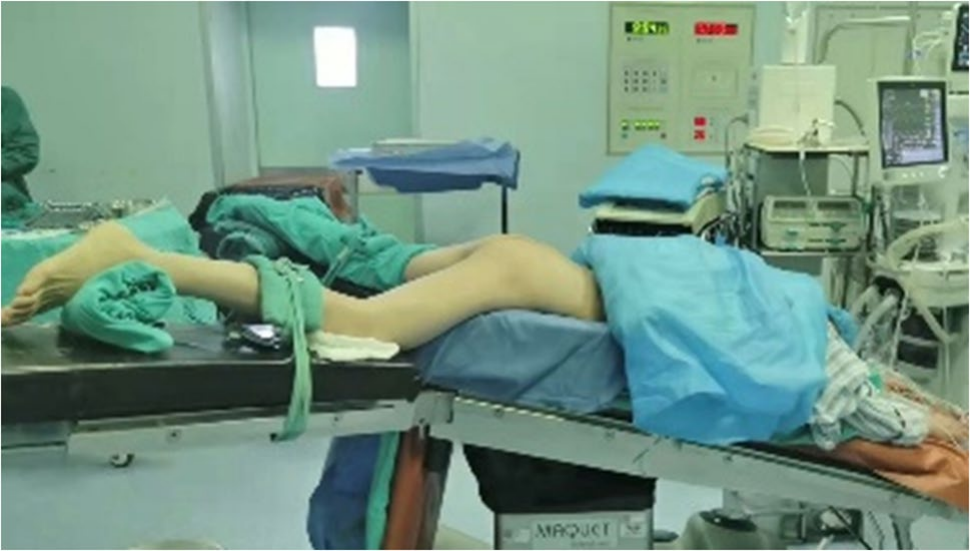
The patient takes a prone split leg position

**Fig. 2:**
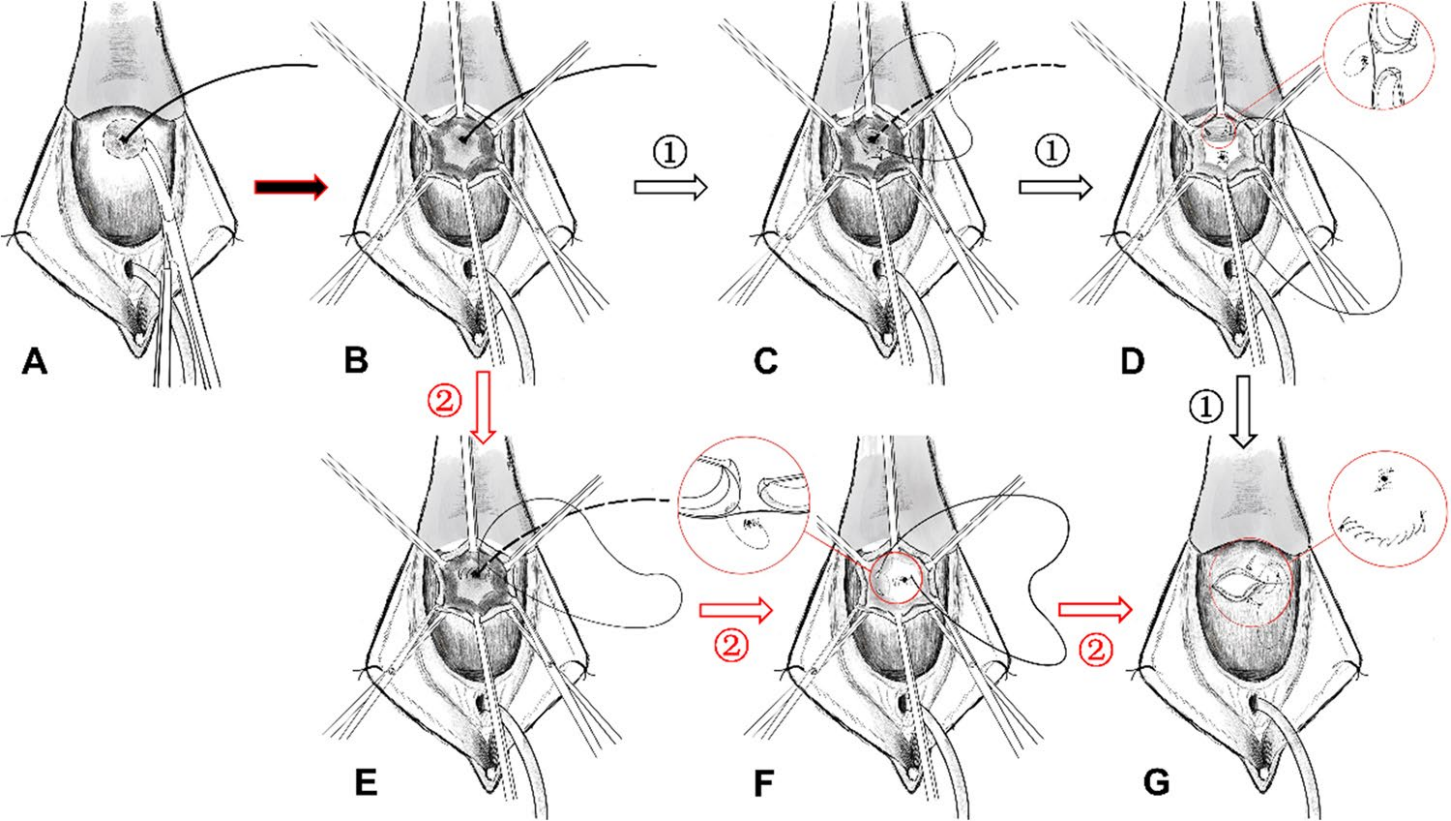
**A** A circumferential incision was made around the fistula opening at least 0.5 cm from the fistula opening. **B** The vaginal flap around the fistula was freed by at least 2–3 cm. **C**, **E** The fistula was sutured continuously. **D**, **F** The fistula was completely suspended and buried behind the apical/lateral free flap. **G** The vaginal mucosa was misaligned and closed. *1* Transferring the apical free flap, *2* transferring the lateral free flap

After general anesthesia, the patient took a prone split leg position (Fig. 1). Both sides of the labia were retracted with suture to optimize vaginal exposure. Vaginal hooks were used to fully expose the anterior vaginal fistula for a good surgical field of view. After inserting the 16-F catheter, the bladder was injected with light iodine fluid to flush the fistula, and then a guidewire was placed from the fistula into the bladder. After infiltrating the vaginal edges of the fistulous tract with saline adrenaline mixture (1:1,000), we made a circumferential incision at least 0.5 cm from the fistulous opening (Fig. 2A). The vaginal flap around the fistula was freed by at least 2 to 3 cm (Fig. 2B). We sutured the fistula continuously using a 3–0 absorbable suture, and ensured that sutures passed through the bladder muscle layer without crossing the mucous membrane (Fig. 2C, E). The distal suture of the fistula was continuously sutured to the apical flap, so that the fistula was completely suspended and buried behind the apical/lateral free flap (Fig. 2D, F). The vaginal mucosa was misaligned and closed with 2–0 absorbable sutures (Fig. 2G). We slowly injected 150 to 200 ml of diluted methylene blue solution through the catheter to observe whether there was fluid leaking from the anterior wall of the vagina. The vagina was filled with iodophor yarn strips, and the operation was completed. All surgeries were performed by the same surgeon.

### Postoperative follow-up

The vaginal suture was removed the day after surgery and the Foley catheter drainage was maintained for 4 weeks. During this period, M-blockers were taken orally to reduce bladder spasms, and estrogen ointment was routinely applied daily in the vaginal cavity. Sexual activity was avoided for 3 months after surgery. The criteria for successful repair were no vaginal leakage in the 12–36 months after surgery, and no fistula on cystoscopic and vaginal examination. Follow-up ranged from 12 to 36 months (average 18 months) to observe recurrence and complications. Subjective assessment of the success of the surgery was performed using the Perception Global Impression of Improvement (PGI-I) questionnaire, which was a self-administered assessment questionnaire with seven questions, with answers ranging from worse to best.

## Results

A total of 37 patients in the group underwent transvaginal flap transfer coverage to repair a bladder–vaginal fistula. The results and complications are shown in Table 2. Thirty patients underwent repair of the fistula by transferring an apical free flap, 7 patients underwent repair of the fistula by transferring a lateral free flap. The operation time ranged from 30 to 45 min (average 37.6 min). Intraoperative blood loss was 2 to 10 ml (average 5.2 ml). The length of hospital stay was 3–5 days (average 4 days). The Foley catheter was removed 4 weeks after surgery, and the follow-up period was 12–36 months (average 22.8 months). Among the 37 patients, 34 cases of primary complete healing, the success rate reached 91.89% without recurrence and complications (the Clavien–Dindo classification of surgical complications was used and the EAU guideline recommendations were followed). There were 3 cases of recurrent leakage of urine, cystoscopy showed that the fistula was significantly reduced, and all were cured after secondary repair by the same surgical method without complications. All the patients fell into the categories of either "much better" (PGI-I: 21.6%) or "very much better" (PGI-I: 78.4%) in the follow-up.

**Table 2:** Results and complications

	Value
The timing of surgical repair(week)	
<6	8 (21.6%)
>6	29 (78.4%)
Operation time (min)	37.6 (30–45)
Intraoperative blood loss (ml)	5.2 (2–10)
Hospitalization time(days)	4(3–5)
Follow-up period (months)	22.8 (12–36)
Complication	0
Transferring the apical free flap	30 (81.1%)
Transferring the lateral free flap	7 (18.9%)
PGI-I	
Much better	8 (21.6%)
Very much better	29 (78.4%)
Outcome	
Success	34 (91.89%)
Recurrence	3 (8.11%)

All were cured after secondary repair by the same surgical method

*PGI-I* Perception Global Impression of Improvement

## Discussion

A majority of VVFs require surgical treatment to cure. There is no consensus on the timing of surgical repair [6]. It is generally considered that the most ideal early repair time is within 72 h of injury, because the surrounding tissue is fresh. Another reason for early repair is that there is no edematous adhesion and no tension during surgical separation and suturing. But most fistulas are detected after several days or weeks of injury, and should be delayed for 3 to 6 months until the fistula resolves, inflammation improves, and there is no infection. However, some researchers have also suggested that regardless of the condition of the tissue around the fistula, it is more appropriate to delay surgical repair for 4–6 weeks [7]. Early surgical repair success rates have been reported to be as high as 100% within 6 weeks of the diagnosis of VVF, with no significant difference compared with delayed surgical repair success rates [8, 9]. Eight of the 37 patients confirmed in our group underwent early repair within 6 weeks.

No matter which surgical method is used, the key to the cure rate of VVF is to fully dislocate the fistula and the incision to prevent infection and formation of a hematoma, to ensure the blood supply of the fistula, and to minimize complications such as dyspareunia (vaginal shortening, contracture) after surgery. Exposure and layer separation are essential to the surgical technique.

There is currently no uniform treatment guideline for VVFs [10]. The path of surgical repair includes simple transvaginal, transabdominal, transvesical, and multiple approaches to combined repair [11]. The choice of surgical modality depends not only on the location, size, number, and vaginal conditions of the fistula, but also on the surgeon's clinical experience and the patient's choice [12]. Studies have shown that nearly three-quarters of VVFs can be repaired vaginally, with a success rate comparable with transabdominal path repair without significant differences [13]. But, owing to its short surgery and hospital stays, transvaginal repair is significantly more cost-effective than transabdominal repair [14]. Angioli et al. [11] argues that transvaginal repair should be considered first for any fistula of etiology unless there are obvious contraindications to the vaginal approach, such as a highly complex fistula and difficulty exposing vaginal stenosis. Recurrent fistula, refractory fistula, and radiation fistula are not absolute contraindications. Transvaginal repair has the advantages of short surgical time, less intraoperative bleeding, short hospitalization time, fast postoperative recovery, strong repeatability, etc. Besides, it is minimally invasive and not limited by the time of postoperative recurrence and re-repair. Transvaginal repair is currently increasingly valued and favored, but owing to a narrow vaginal space, difficult operation, and higher requirements for separation skills, the development of this surgical path is still limited.

At present, the common surgical techniques for transvaginal repair are:

1. Latzko surgery: a simple and effective surgical method, which requires first annular resection of the vaginal mucosa around the fistulous tract at 1 cm or 1.5 cm. At the same time, the vaginal fistula can be removed or not, and then the bladder and the intervaginal fascia of the bladder are sutured in layers and the fistula was inverted towards the bladder without entering the bladder mucosa. Finally the vaginal mucosa is completely sutured [15, 16]. As the surgical operations do not enter the bladder, the risk of urinary tract infection is reduced, while avoiding bladder, ureteral and urethral injuries, an intraoperative bleeding is relatively low. However, it has been reported that the procedure may result in postoperative urinary incontinence, vaginal shortening, and difficulty having intercourse [17, 18]. Dorairajan et al. [19] reported that no such postoperative complications have been found. For apical VVF, Luo and Shen [20] used modified Latzko surgery in a large cross-sectional and observational study of 108 patients. Considering that apical VVF are often hidden or have adhered to previous surgical scars at the apical vagina, the Latzko technique is combined with vaginal occlusion, multi-layer sutures are deeply buried in the sinus tract, and the vaginal apex is reconstructed. The success of the first phase of surgery repair is 91.89%, and there are no obvious complications.
2. Layered closure: for a simple, small fistula, the layered closure method can achieve a very high first-stage repair rate, and there is a certain similarity to Latzko surgery, which removes part of the bladder mucosa, followed by full-stage excision of the fistula, a layered tile-like suture to close the bladder, and finally a suture to close the vaginal wall. Unlike Latzko surgery, this procedure removes part of the bladder mucosa [21].

This surgery is also a modified Latzko procedure that uses only vaginal free flaps without removing the fistulous tract. Current thinking argues against fistulous tract excision to reduce the size of the fistula as much as possible, to minimize bleeding, to avoid damage to the ureter, and to retain the fibrous ring to ensure tension-free closure. Studies have shown that fistula diameter ≥1 cm and repeated transvaginal repair are independent prognostic factors for failure in transvaginal repair [22].

This study used the transvaginal flap transfer covering method to repair VVF, and summarized the experience as follows:

1. The prone split leg position was adopted, and the fistula was placed below, which was more in line with the operating habits of the surgeon. At the same time, with the help of the vaginal hook pulling up, it is more conducive to exposure, which is obviously better than with the lithotomy position.
2. Do not remove the fistula to avoid enlarging the fistula, reduce bleeding, avoid ureteral damage, reduce the incidence of bladder spasm, not affect the success rate of repair, and make it easier to repair again.
3. The vaginal flap around the fistula is sufficiently free and the fistula is moved up.
4. After the first layer of the fistula is sutured, the fistula flap is transferred, so that the fistula is directly attached to the vaginal flap, which can prevent hematoma or urine leakage around the fistula, leading to infection, poor healing, and failure of the surgery.
5. The fistula is completely embedded under the flap, close to the vaginal wall and away from the incision suture surface, which effectively reduces the recurrence rate of the VVF.
6. Advantages of transferring a lateral free flap: if the patient is sexually active and the vagina is not long enough, the use of Latzko surgery may easily lead to vaginal shortening, but transvaginal fistula suspension by transferring the lateral flap can ensure the vaginal length, especially for cases with a relatively low fistula.
7. This surgical approach is suitable for VVFs of any location, not just apical fistulas.
8. The use of estrogen ointment and postoperative oral drugs to reduce bladder spasm can effectively improve the success rate of surgery.

Analysis of the causes of failure cases was as follows:

1. One case had intraoperative sutures through the urinary catheter, and was forced to have the sutures cut early, resulting in surgical failure.
2. Preoperative cystoscopy or intraoperative methylene blue dye test did not find another small fistula, so urinary leakage occurred after surgery.
3. Other causes: may be accompanied by overactive bladder and persistent bladder hypertension, leading to poor healing and urinary leakage.

Our research shows that the surgical treatment of VVF by transvaginal flap transfer coverage is feasible and effective, and the first-stage repair is successful in 91.89%. The effect is long-lasting without obvious complications, which is worth promoting and applying in the clinic. Transferring the lateral free flap can maintain vaginal length to avoid vaginal shortening. However, the choice of surgical modalities depends on the experience of the surgeon and there may be a selection bias. Besides, this study is a retrospective cohort study, and there is a lack of randomized controlled studies of large datasets with different surgical modalities.

## Conclusion

Transvaginal flap transfer coverage is a safe and effective surgical method for repairing VVF. The prone split leg position can better increase exposure. The fistula away from the incision suture is the key to the success of the operation. Transferring a flap can effectively improve the cure rate of VVF. Transferring the lateral free flap can maintain vaginal length to avoid vaginal shortening. Combining preoperative and postoperative drug therapy can effectively improve the success rate of surgery.

## Supplementary information

Below is the link to the electronic supplementary material.

Supplementary file1 (MP4 42377 kb)

## Data Availability

Data will be provided upon request to the authors.

## Abbreviations

VVF: Vesicovaginal fistula
OAB: Overactive bladder

## References

[1] Goodwin WE, Scardino PT (1980). Vesicovaginal and ureterovaginal fistulas: a summary of 25 years of experience. J Urol.

[2] Marks P, Kluth LA, Lang IJ (2020). Vesicovaginal fistulas: diagnosis and surgical management. Urologe A.

[3] Hillary CJ, Osman NI, Hilton P (2016). The aetiology, treatment, and outcome of urogenital fistulae managed in well- and low-resourced countries: a systematic review. Eur Urol.

[4] Wall LL (2006). Obstetric vesicovaginal fistula as an international public-health problem. Lancet.

[5] Malik MA, Sohail M, Malik MT (2018). Changing trends in the etiology and management of vesicovaginal fistula. Int J Urol.

[6] Lee D, Zimmern P (2019). Vaginal approach to vesicovaginal fistula. Urol Clin North Am.

[7] Blandy JP, Badenoch DF, Fowler CG (1991). Early repair of iatrogenic injury to the ureter or bladder after gynecological surgery. J Urol.

[8] Badenoch DF, Tiptaft RC, Thakar DR (1987). Early repair of accidental injury to the ureter or bladder following gynaecological surgery. Br J Urol.

[9] Moriel EZ, Meirow D, Zilberman M (1993). Experience with the immediate treatment of iatrogenic bladder injuries and the repair of complex vesico-vaginal fistulae by the transvesical approach. Arch Gynecol Obstet.

[10] Zambon JP, Batezini NS, Pinto ER (2010). Do we need new surgical techniques to repair vesico-vaginal fistulas?. Int Urogynecol J.

[11] Angioli R, Penalver M, Muzii L (2003). Guidelines of how to manage vesicovaginal fistula. Crit Rev Oncol Hematol.

[12] Gerber GS, Schoenberg HW (1993). Female urinary tract fistulas. J Urol.

[13] Rajamaheswari N, Chhikara AB, Seethalakshmi K (2012). Trans-vaginal repair of gynecological supratrigonal vesicovaginal fistulae: a worthy option!. Urol Ann.

[14] Warner R, Beardmore-Gray A, Pakzad M (2020). The cost effectiveness of vaginal versus abdominal repair of vesicovaginal fistulae. Int Urogynecol J.

[15] Ansquer Y, Mellier G, Santulli P (2006). Latzko operation for vault vesicovaginal fistula. Acta Obstet Gynecol Scand.

[16] Kieserman-Shmokler C, Sammarco AG, English EM (2019). The Latzko: a high-value, versatile vesicovaginal fistula repair. Am J Obstet Gynecol.

[17] Singh V, Sinha RJ, Sankhwar SN (2011). Transvaginal repair of complex and complicated vesicovaginal fistulae. Int J Gynaecol Obstet.

[18] Farahat YA, Elbendary MA, Elgamal OM (2012). Application of small intestinal submucosa graft for repair of complicated vesicovaginal fistula: a pilot study. J Urol.

[19] Dorairajan LN, Khattar N, Kumar S (2008). Latzko repair for vesicovaginal fistula revisited in the era of minimal-access surgery. Int Urol Nephrol.

[20] Luo DY, Shen H (2019). Transvaginal repair of apical vesicovaginal fistula: a modified Latzko technique—outcomes at a high-volume referral center. Eur Urol.

[21] El-Azab AS, Abolella HA, Farouk M (2019). Update on vesicovaginal fistula: a systematic review. Arab J Urol.

[22] Yang Y, Chen YK, Che XY (2021). Prognostic factors for failure of transvaginal repair of vesicovaginal fistula: a nested case-control study. Beijing Da Xue Xue Bao Yi Xue Ban.

